# Adenocarcinoma of Sigmoid Colon Diagnosed in Pregnancy: A Case Report

**DOI:** 10.7759/cureus.9491

**Published:** 2020-07-31

**Authors:** Metlapalli Venkata Sravanthi, Sharmil Suma Kumaran, Abhinav Palle, Padmaja Bojanapally

**Affiliations:** 1 Internal Medicine, The Wright Center for Graduate Medical Education, Scranton, USA; 2 Student, Abington Heights High School, Scranton, USA; 3 Oncology, Hematology & Oncology Associates of Northeastern Pennsylvania, Scranton, USA

**Keywords:** colorectal carcinoma, pregnancy, crc-p

## Abstract

With more women getting pregnant at later ages than in the past, the incidence of malignancies in pregnancy is on the rise. Common malignancies of pregnancy are melanoma, breast cancer, cervical cancer, lymphomas, and leukemias. Colorectal carcinoma is rare in pregnancy, with an estimated incidence of 1 in 13',000 cases. We describe such a case of colorectal carcinoma in pregnancy (CRC-p), in a 31-year-old patient. She presented in the 21^st^ week of her second pregnancy with constipation of two weeks duration despite appropriate medical management. This prompted further evaluation with abdominal imaging revealing partial small bowel obstruction of unclear etiology. She was treated surgically with subtotal colectomy with ileostomy. Pathologic evaluation revealed Stage III B: pT3N2a adenocarcinoma with mucinous features of the sigmoid colon with lymph node metastases. Adjuvant FOLFOX chemotherapy was started in the third trimester and was continued postpartum for a total of 12 cycles. She is doing well, and ileostomy reversal is being planned at the time of writing this.

Advancing maternal age is a significant risk factor for CRC-p. Common presenting symptoms in CRC-p include bleeding per rectum, abdominal pain, vomiting, and constipation. The frequent occurrence of many of these symptoms, as well as risks and restrictions associated with diagnostic modalities such as computed tomography scan and colonoscopy during pregnancy, makes the diagnosis challenging. Colonoscopy, followed by pathology evaluation, remains the standard diagnostic method in CRC-p. Management of CRC-p is determined by multiple variables such as the stage of the disease, gestational age, and most importantly, patient wishes. Surgical resection is performed following the diagnosis if the gestational age is less than 20 weeks and delayed until after delivery if gestational age is above 20 weeks. 5-fluorouracil based chemotherapy regimens are used in second and third trimesters, in patients with stage III CRC-p. Prognosis has been reported variably. Despite advanced stages at presentation, most of the studies indicate a similar prognosis compared to CRC in the non-pregnant population. Two-year survival was found to be 64.4% in one case series.

## Introduction

Most frequent cancers associated with pregnancy are melanoma, breast cancer, cervical cancer, lymphomas, and leukemias, in the order of decreasing frequency [1]. Although colorectal carcinoma (CRC) is the second most common cancer in women worldwide, colorectal carcinoma in pregnancy (CRC-p) is rare and is often associated with poor prognosis. We describe a case of stage III adenocarcinoma of sigmoid colon diagnosed during the second trimester of pregnancy.

## Case presentation

**Figure 1:**
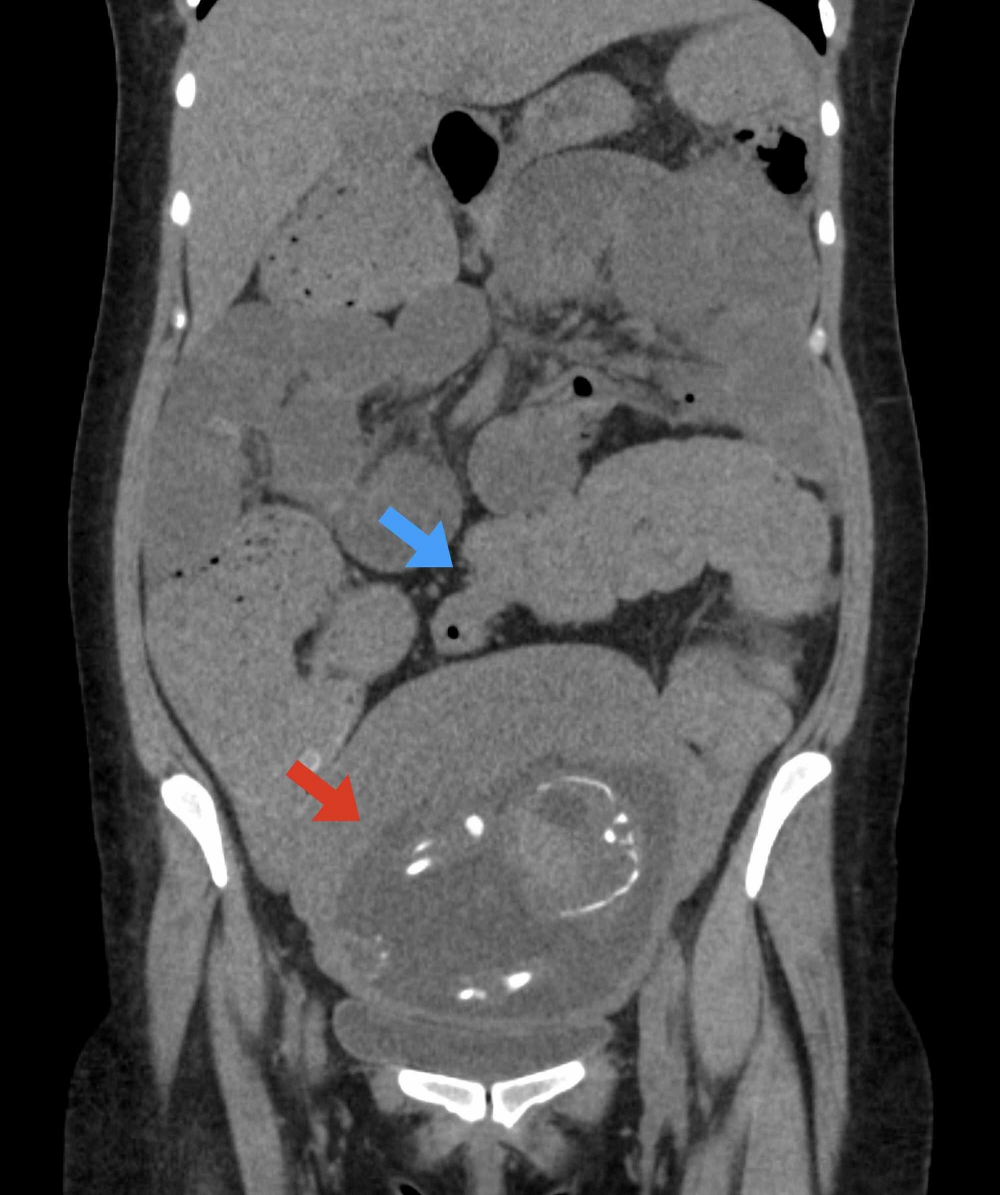
Coronal reconstruction from CT scan of the abdomen and pelvis demonstrating colonic distention with abrupt transition to complete collapse in the region of the sigmoid colon (blue arrow) and gravid uterus (red arrow)

**Figure 2:**
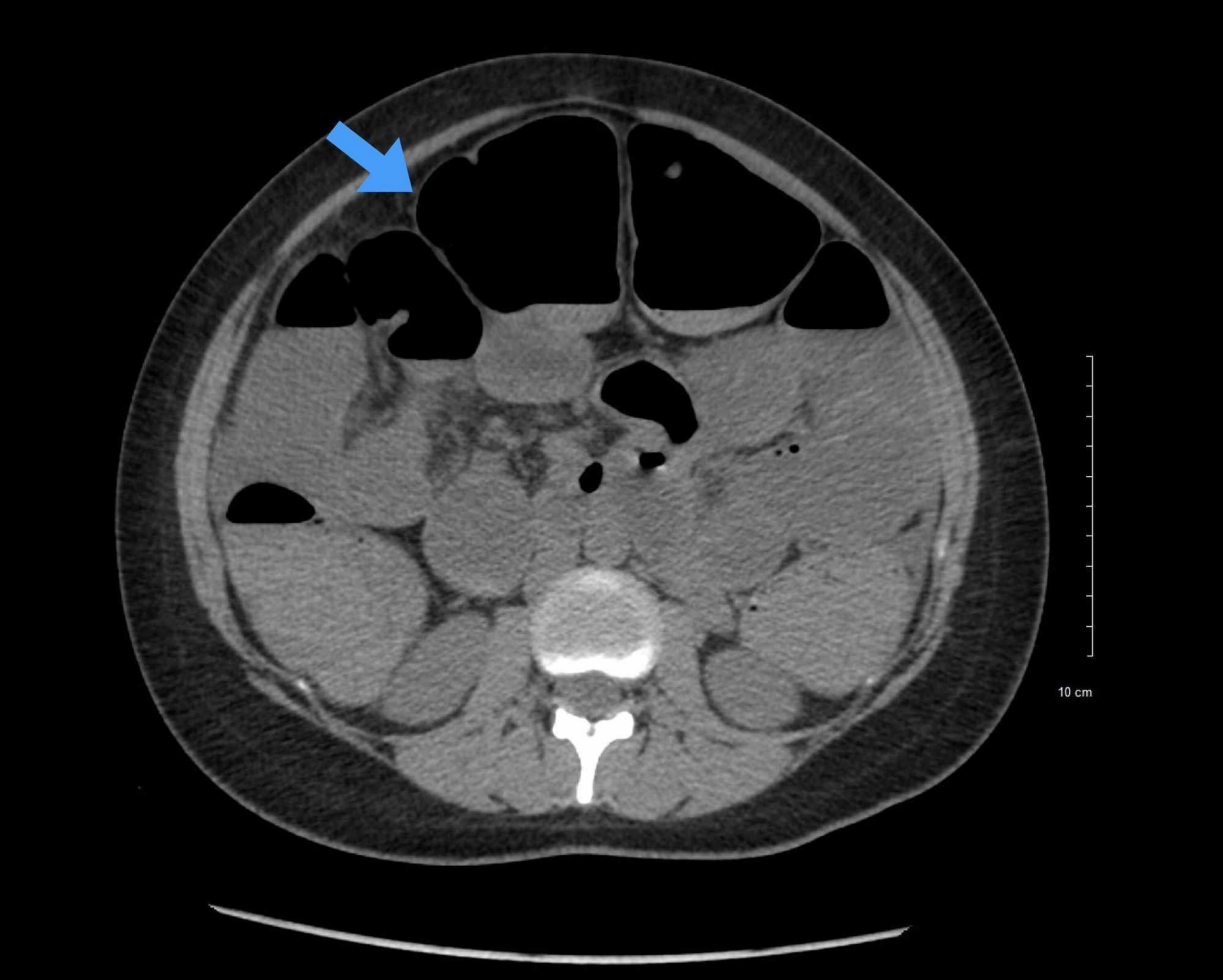
Axial slice from CT scan of the abdomen demonstrating dilated small bowel loops with air-fluid levels

A 31-year-old female gravida 2 para 1 (G2P1) in her 21st week of pregnancy was evaluated in the emergency room (ER) for constipation of 10-day duration. She also endorsed abdominal pain, nausea, and non-bloody vomiting. She denied any hematochezia or melena. The only risk factor for her constipation was her pregnancy. She was not on any medications that could cause constipation, including narcotics, nor did she have any recent surgery or thyroid disorders. On initial evaluation, her vital signs were within normal limits. The physical examination was consistent with her pregnancy. An abdominal sonogram demonstrated a single live intrauterine pregnancy and no other abdominal findings of note. She was prescribed stool softeners and osmotic laxatives and discharged. She returned to the ER the next day with a lack of relief despite using those medications. This time abdominal distention appeared more pronounced, and palpation of the abdomen demonstrated diffuse tenderness. No other changes in clinical or laboratory evaluation were noted. She was reassured and discharged home with the addition of metoclopramide and a stimulant laxative. She presented for a third time to the ER the day after, with worsening vomiting and ongoing constipation. Her vital signs were unremarkable. Pertinent laboratory findings were neutrophilic leukocytosis (total leukocyte count 13.3 x 10^9^/L; reference range 4.5-10 x 10^9^/L) and baseline anemia (hemoglobin 8 g/dL; reference range 12.5-15.5 g/dL). Abdominal radiography revealed dilated small bowel measuring up to 5.4 cm in diameter and stool throughout the colon suspicious for small bowel obstruction. A CT scan of the abdomen showed marked colonic distention with a transition point between distended and collapsed bowel in the region of the sigmoid in addition to the dilated small bowel, concerning for mechanically significant colonic obstruction (Figures 1, 2). No obvious masses were identified. There were ascites and mesenteric edema. She was admitted for further evaluation and management. After multiple attempts, a nasogastric tube was inserted. At this point, general surgery and obstetric services were consulted. After reviewing the risks and benefits of operative and nonoperative management and a possibility of fetal loss since the fetus was still pre-viable, a decision to proceed with the former was made. A subtotal colectomy with ileostomy was done the next day. She was started on intravenous fluids and broad-spectrum antibiotics perioperatively.

Postoperatively, indomethacin suppositories were given for 24 hours as a precaution to quiesce the uterus, should uterine activity occur. Fetal viability was confirmed with fetal heart tracing and obstetric ultrasound. Analysis of peritoneal fluid demonstrated no evidence of malignancy. However, a culture of the fluid grew staphylococcal species, which was managed with antibiotics tailored per sensitivity report. Her condition improved, and she started tolerating oral feeds.

Pathologic evaluation of the total abdominal colectomy specimen revealed adenocarcinoma with mucinous features of the sigmoid colon measuring 3.5 cm in the largest dimension and six positive lymph nodes out of 16. Pathologic classification was assigned as stage III B: pT3N2a (pTNM, AJCC 8th Edition) [2]. The histologic grade was determined to be moderately differentiated (G2). Immunohistochemical stains for mismatch repair were negative. A positron emission tomography (PET) CT scan or CT with contrast was not done due to potential fetal adversities. An adjuvant chemotherapy with FOLFOX (folinic acid “FOL,” 5-fluorouracil “F,” and oxaliplatin “OX”) regimen was chosen to prevent recurrence and to prolong progression-free survival. The chemotherapy was deferred until the third trimester as the risk for adverse effects was thought to be minimal in the third trimester, and benefits outweighed the risks of adverse effects. She was eventually discharged home.

She was followed up regularly as an outpatient. She was started on the FOLFOX regimen in the third trimester as planned. Her pregnancy continued uneventfully, resulting in the birth of a healthy child via normal vaginal delivery in the 39th week. She received five cycles of chemotherapy before delivery. During the hospitalization for confinement, she was noted to have stable mild anemia and thrombocytopenia, possibly due to chemotherapy. The FOLFOX regimen was resumed after delivery. A CT scan of the abdomen with contrast obtained one month after childbirth revealed no evidence of metastasis, but an 11 mm para-aortic lymph node. She completed 12 cycles of FOLFOX with the development of adverse effects, including anemia, thrombocytopenia, and peripheral neuropathy. She remained healthy and maintained a functioning ileostomy. At the time of writing this, an ileostomy reversal is being planned.

## Discussion

The most common cancers associated with pregnancy are melanoma, breast cancer, cervical cancer, lymphomas, and leukemia, most of which have a peak incidence in the reproductive age group. CRC is usually more common above the age of 50. However, CRC incidence among individuals under the age of 50 in the United States steadily increased at a rate of 2.1% per year from 1992 to 2012 [3]. It has continued the trend since then. Also, the average age of first-time mothers increased 4.9 years from 1970 to 2014, from 21.4 to 26.3 years [4]. With advancing maternal age, malignancies in pregnancy including CRC-p are becoming increasingly frequent. The estimated incidence of CRC-p is 1 in 13,000 cases [5]. Although the incidence of CRC-p is very low, it parallels the trend of the increase in the incidence of CRC in younger individuals.

Age is a significant risk factor for CRC, and the incidence increases significantly with advancing age. In an extensive systematic review of 41 cases of CRC-p by Bernstein et al., the mean age at presentation was found to be 31 (range 16-41) years [5]. Another review by Pellino et al. had similar findings, with a median age at presentation of 32 (range 17-46) years [6]. Apart from the risk factors for sporadic CRC such as age, African American inheritance, high red meat consumption, obesity, etc., no specific risk factors for CRC-p has been identified. Colon cancer caused by familial adenomatous polyposis has also been reported in pregnancy [7].

Upon review of previously reported cases, the involvement of colon and rectum have been seen in comparable frequencies. Common presenting symptoms include bleeding per rectum, abdominal pain, vomiting, and constipation. There were bowel obstruction and perforation in some cases [6]. Cases presenting with intussusception have also been reported [8]. The diagnosis of CRC-p is quite challenging. Since the symptoms such as abdominal pain and constipation are common in pregnancy, they can be easily overlooked. Also, diagnostic workup such as imaging and colonoscopies are not often pursued in the pregnant population due to potential risks to the fetus. Even when the workup is done, pregnancy can interfere with the sensitivity and specificity of these diagnostic tests. All these can result in a delay in diagnosis and advanced stages at presentation. The Duke stage at presentation was B or above in all the cases in one series involving 41 cases [5].

The diagnosis depends on the nature of the presentation. In our case, the patient presented with small bowel obstruction, which was managed with emergent surgical intervention. Subsequent pathological evaluation of the surgical specimen led to the diagnosis. Colonoscopies remain an invaluable tool for the determination of CRC in the pregnant population as well. Colonoscopy during pregnancy is of low risk for mother and child in all three trimesters of pregnancy [9]. A retrospective study determined that colonoscopies are relatively safe during the second trimester, without substantial fetal risks [10]. Potential complications of colonoscopy specific to pregnancy include placental abruption, fetal injury, or loss. The imaging modality of choice for the abdomen and pelvis in pregnancy is MRI. The safety of contrast is not proven in pregnancy and is better avoided [11]. CT scan of the abdomen and pelvis can also be performed when a fetal radiation threshold of 100 milligrays is maintained [12,13]. CT scan of other regions for staging purposes can be done with adequate abdominal shielding. Abdominal ultrasonography is also a safe diagnostic tool to evaluate metastatic disease, especially in the liver. Endoluminal ultrasonography can aid in confirming the staging of rectal lesions. Carcinoembryonic antigen (CEA) is elevated but within the normal range during pregnancy. Elevated CEA above normal is useful as a prognostic factor and should be periodically monitored.

There are no incidences of the fetal spread of malignancy noted in any of the reported cases of CRC-p. The fetal risk from the malignancy itself is minimal. Placental metastasis is extremely rare and has been reported only once [14]. The incidence of ovarian metastasis is said to be as high as 25% in pregnancy, compared to 5% in non-pregnant female population [15]. Management of CRC-p depends on many factors such as stage, nature of the presentation, disease progression, gestational age, and patient wishes. The mainstays of treatment are surgical resection and chemotherapy. The current consensus on the timing of surgery is immediately after the diagnosis if the gestational age is less than 20 weeks, and one to two weeks after delivery if the gestational age is more than 20 weeks [16]. Despite frequent metastasis to ovaries, routine oophorectomy is not recommended. Premature delivery and low birth weight have been reported to occur twice as numerous following surgeries compared to the general pregnant population. Chemotherapy is recommended in patients in stage III. Chemotherapy is avoided in the first trimester due to harmful effects on organogenesis. 5-fluorouracil-based chemotherapy regimens are frequently utilized due to its relative safety in the second and third trimesters, with a lower risk of abortion and fetal malformation [1,17]. However, stillbirth, intrauterine growth restriction, and fetal toxicities are found to occur more frequently in patients who received chemotherapy after the first trimester [18]. Hence the decision-making related to chemotherapy whenever appropriate, should be left primarily to the patient herself. Radiotherapy is not useful in colon cancer and is especially avoided in pregnancy [1]. In the first half of pregnancy, abortion is offered, should the patient no longer wish to proceed with the pregnancy. The mode of delivery is determined based on obstetric parameters and is not affected by the diagnosis of cancer itself. The approach to CRC-p is multimodal and involves teamwork, including the oncologist and obstetrician.

The prognosis of CRC-p has been reported variably. Many systematic reviews reported similar prognosis when compared to the general population, despite advanced stages at presentation [5,19]. One case series indicated a significantly poorer prognosis compared to the general population [20]. The median survival was 42 (range 0-120) months in one systematic review [6]. The overall two-year survival was found to be 64.4% in another series [19].

## Conclusions

Colorectal carcinoma in pregnancy is a devastating diagnosis, adding immense physical and emotional stress to the expectant mother and family. Attention to persistent non-specific symptoms in pregnancy, early diagnosis, and therapy driven by shared decision making are vital in addressing this unfortunate situation.
